# Pathology of African Swine Fever in Reproductive Organs of Mature Breeding Boars

**DOI:** 10.3390/v15030729

**Published:** 2023-03-11

**Authors:** Julia Sehl-Ewert, Virginia Friedrichs, Tessa Carrau, Paul Deutschmann, Sandra Blome

**Affiliations:** 1 Department of Experimental Animal Facilities and Biorisk Management, Friedrich-Loeffler-Institut, Südufer 10, 17493 Greifswald-Insel Riems, Germany; 2 Institute of Diagnostic Virology, Friedrich-Loeffler-Institut, Südufer 10, 17493 Greifswald-Insel Riems, Germany

**Keywords:** African swine fever, boar, domestic pig, reproductive organs, testis, epididymis, vesicular gland, prostate, bulbourethral gland, pathology, macroscopy, histopathology, sperm

## Abstract

African swine fever (ASF) is a severe, globally important disease in domestic and wild pigs. The testing of alternative transmission routes has proven that the ASF virus (ASFV) can be efficiently transmitted to sows via semen from infected boars through artificial insemination. Boars intramuscularly inoculated with the ASFV strain “Estonia 2014” showed grossly and microscopically visible changes in the testis, epididymis, prostate, and vesicular gland. The gross lesions included hemorrhages on the scrotum, testicular membranes, and parenchyma; edema; hydroceles; and proliferations of the tunica vaginalis. Histopathologically, vasculitis and perivasculitis was detected in the testis and epididymis. Subacutely infected animals further revealed a degeneration of the testicular and epididymal tubules, pointing to the destruction of the blood–testis and blood–epididymis barriers upon disease progression. This was confirmed by evidence of semen round cells and sperm abnormalities at later time points after the infection. The histopathology was associated with the presence of viral DNA and the infectious virus, and in a limited amount with viral antigens. In most scenarios, the impact of these changes on the reproductive performance and long-term persistence of the virus is probably negligible due to the culling of the animals. However, under backyard conditions and in wild boar populations, infected males will remain in the population and the long-term fate should be further evaluated.

## 1. Introduction

With its cross-continental spread, African swine fever (ASF) poses a significant threat to global pig farming. It is a severe, and in most cases lethal, disease in domestic pigs and wild boars, and is characterized by a high fever and disseminated hemorrhages [1]. On-farm trials of a recently licensed vaccine in Vietnam give hope for a method to combat the disease [2]. The incubation period of the ASF varies between 3 and 19 days [3], but is roughly 4 days based on experimental observations [4]. In the initial phase of infection, animals show rather unspecific clinical signs such as a fever or a lack of appetite, which do not necessarily indicate ASF at first [1]. The virus is transmitted by direct and indirect contact. Direct transmission occurs via body fluids such as nasal and oral secretions, feces, and primarily via blood [5]. Decades ago, Thacker et al. [6] postulated that ASFV-contaminated semen could also be a threat for the transmission of the disease to healthy animals, but until recently, there was no evidence to support this hypothesis.

Recent studies have already proven that infectious ASFV and the viral genome are present in male genitals and in the postmortem-sampled epididymal sperm of mature boars and adolescent wild boars infected with ASFV isolates of different genotypes [7,8]. In addition, the lesions detected in male genitals support ASFV pathogenesis in the reproductive tract [7]. While gross changes were absent in the acute stage on days seven to eight post-infection, histopathological alterations were already detectable and included vasculitis and perivasculitis, apoptosis/necrosis of the testicular stromal cells, and mild infiltration of the smooth muscle layer of the epididymal ducts. ASFV mRNA was found in single halo cells of the epididymal duct epithelium, posing a risk that ASFV could overcome the blood–epididymis barrier. However, at that time, no further histopathological changes could identify the source of ASFV-positive epididymal sperm, and the destruction of the blood–testis or blood–epididymis barriers could not be demonstrated pathomorphologically. However, the limitation of these studies was the high virulence of the ASFV strains, which resulted in acute severe disease with an early humane endpoint [7,8].

Of note, very recently, Friedrichs et al. [9] demonstrated that the efficient transmission of ASFV is possible via the introduction of semen from infected breeding boars to sows by artificial insemination. The authors collected semen from breeding boars infected with the moderately virulent ASFV strain “Estonia 2014” and inseminated gilts. Fifty percent of the sows were ASFV-positive by seven days post-insemination, whereas on day 35, all animals were positive. A pregnancy was detected in 13 of the 14 sows, of which 12 sows aborted or resorbed.

The aim of the present study was to deepen our knowledge of ASF pathogenesis and pathology, particularly with regard to the reproductive tract; to determine the progression of pathological changes; and to identify the passage of ASFV into semen in the subacute stage of ASF.

## 2. Materials and Methods

### 2.1. Study Design

In accordance with the current German Animal Welfare regulations, the animal experiment was approved by the competent authority (Landesamt für Landwirtschaft, Lebensmittelsicherheit und Fischerei Mecklenburg-Vorpommern (LALLF M-V)) under reference number 7221.3-1-071/21.

The initial objective of this study was to assess the detectability of ASFV infections in male breeding animals, especially in the early phase of the disease, and to provide baseline data on the transmissibility via artificial insemination. Samples from this recently published study [9] were archived to allow for more detailed analyses into the pathology and pathogenesis in male animals.

Thus, we were able to continue our previous work [7] and set out to study the pathomorphological changes to the reproductive tract in intact boars after infection with a moderately virulent ASFV strain in more depth.

### 2.2. Cells and Virus

The inoculum was prepared from a spleen derived from a pig of a previous trial infected with the moderately virulent ASFV strain “Estonia 2014”. Details on the preparation procedure and titration of the spleen suspension are described elsewhere [9].

### 2.3. Animal Experiment

All details of the experimental design of the initial study can be found in the recently published work describing the transmission of ASFV from infected boars to gilts via artificial insemination [9].

Briefly, a total of four boars were examined in this study, of which two were Large White boars (493/494 days of age at start of trial) and two were Pietrain boars (396 days old) originating from a commercial pig-breeding company (Federal Hybrid Breeding Program, BHZP). Upon arrival, the animals were ear-tagged for unique identification with #1 to #4. The boars were kept individually in a pen with contact to each other. Before inoculation, the animals were allowed an acclimatization period of around one week. Following the natural route of infection, the boars were initially inoculated oro-nasally with 10 mL of a spleen suspension containing approximately 1 × 10^5^ hemadsorbing units with 50% (HAU_50_) per ml of the ASFV strain “Estonia 2014” (gt II). Since no ASFV genome was detectable in the blood at 4 days pi, the animals were again inoculated by the intramuscular route with 1 × 10^4^ HAU_50_ of the same strain to ensure the induction of viremia in each boar. The animals were examined daily and scored for changes in behavior and external appearance using a clinical scoring system with a maximum of 15 points (=humane endpoint) [10]. The animals were euthanized either at the humane endpoint or when clinical signs were intolerable. All boars were subjected to a necropsy.

### 2.4. Sample Collection

As previously described [9], a full necropsy was performed on all boars, and tissue samples from the spleen, hepatogastric lymph node, lung, and liver as well as the male reproductive tract, including the testis, epididymal head, body and tail, prostate, vesicular gland, and bulbourethral gland, were taken for histopathological investigation.

Semen was collected at days −7, −3, −2, −1, 0, 2, 3, 4, 5, 14, and 20 post-IM inoculation.

### 2.5. Pathomorphological Analysis

#### 2.5.1. Gross Pathology and Macroscopic Scoring

All boars underwent a full pathological investigation and detailed gross lesion scoring following the protocol from Galindo-Cardiel et al. [11], with slight modifications as described in [12,13]. In general, the lesions were evaluated based on an ordinal scale from 0 to 3 (normal (0), mild (1), moderate (2), or severe (3)). Tissue samples were fixed in 10% neutral-buffered formalin for at least 3 weeks.

#### 2.5.2. Histopathology and Immunohistochemistry

Formalin-fixed tissue was prepared and processed as described previously [12]. Briefly, fixed tissue samples were embedded in paraffin wax and further processed to 3 µm thin sections. Sections were stained with hematoxylin–eosin (HE) to evaluate the tissue and cellular architecture. ASFV antigens were visualized via avidin–biotin-based immunohistochemical detection using an in-house rabbit polyclonal primary antibody against the major capsid protein p72. The staining procedures are described in a recent work [12].

#### 2.5.3. Semiquantitative Scoring

While the lesions obtained on the HE-stained sections were qualitatively described, the amount of viral antigens was semi-quantitatively scored according to a previously published protocol [12,13]. Histological specimens were scanned using a Hamamatsu S60 scanner and evaluated using NDPview.2 plus software (Version 2.8.24, Hamamatsu Photonics, Shizuoka, Japan). The most affected area (420 × 260 µm) per sample section was scored with score of 0 (no antigen), 1 (1–3 positive cells), 2 (4–15 cells), or 3 (>16 cells). Cells with fine granular cytoplasmic labeling were considered positive, whereas chromogen aggregations without a cellular association were not counted.

### 2.6. ASFV Genome Detection and Virus Isolation

To detect the ASFV genome, DNA was extracted from the reproductive organs, spleen, whole semen, and the semen cell fraction, followed by qPCR. A hemadsorption test (HAT) was performed to detect the infectious virus in the semen, spleen, and reproductive organs. The results of the HAT were indicated as negative, − (all wells negative); weak-positive, + (up to 4 wells positive); positive, ++ (4–8 wells positive); or strong positive, +++ (4–8 wells positive, high rosette counts). Details on the DNA extraction, qPCR, and HAT procedures can be read elsewhere [9].

### 2.7. Semen Processing and Investigation

As described previously [9], semen was diluted with a BTS semen extender (Minitube, Tiefenbach, Germany) and kept at 37 °C to ensure spermatozoa viability. Spermatozoa were counted and evaluated for viability/motility for each collection. To assess the semen quality, spermatozoa were counted using a Neubauer counting chamber and the No./mL were calculated. Additionally, spermatozoa with mobility-hampering abnormalities (e.g., coiled tails, aggregates on tail) were counted and a percentage of the total count was calculated. Slides were kept at 37 °C prior to evaluation to enable a motility assessment. Anomalies in spermatozoa motility (e.g., slow to no movement or moving in circles only) and morphology (defects in head, midpiece, and/or tail) were determined prior to counting. A percentage of the total count was calculated. The viability of infiltrated cells (to exclude aggregates) was assessed by trypan blue staining. Evaluation procedures routinely used for boar studs were employed (BHZP).

## 3. Results

### 3.1. Gross Pathology

All four boars designated as #1, #2, #3, and #4 developed severe clinical signs indicative of ASF and were euthanized when they reached the humane endpoint at days 10, 11, 17, and 25 pi, respectively, according to Friedrichs et al. [9]. The boars were submitted to a necropsy and evaluated in a comprehensive manner according to the adapted protocol from Galindo-Cardiel [11,13]. A macroscopic examination of the animals revealed characteristic findings of ASF infection, although the overall macroscopic pathological changes were less pronounced in boars #3 and #4 than in #1 and #2 due to differences in the time of euthanasia. The details of all organ lesions can be found in Table S1 and Figure S1.

#### Pathology of Male Reproductive Organs

A macroscopic investigation of the male genitals revealed up to severe pathologic changes, as listed in Table 1.

**Figure 1:**
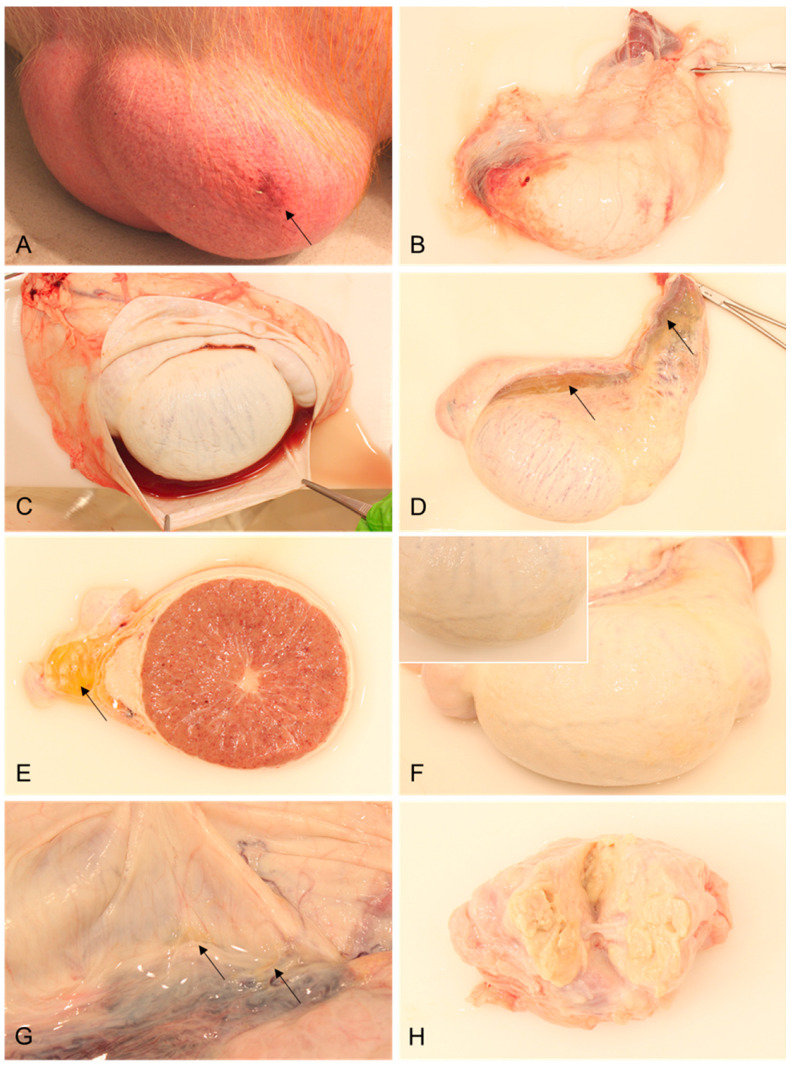
Representative lesions of reproductive organs in mature breeding boars infected with ASFV “Estonia2014”. (**A**) Scrotum with diffusely reddened skin, multifocal pinpoint hemorrhages, and a focal bruise (arrow); (**B**) testis in toto surrounded by hemorrhagic and edematous fascia spermatica externa; (**C**) opened processus vaginalis with a serosanguinous hydrocele; (**D**) testis in toto, with edematous proximal and distal mesorchia (arrows); (**E**) transversal section of the testis and epididymis with severe edema of the distal mesorchium (arrow) and multifocal hemorrhages in the testicular parenchyma; (**F**) testis in toto with mesothelial proliferations (inset: irregular tunical visceral surface with plaque-like lesions); (**G**) opened processus vaginalis with filamentous adhesions (arrow) of the parietal and visceral surface of the tunica vaginalis; and (**H**) cut surface of the prostate, with suppurative inflammation.

The observed external alterations were diffusely reddened scrotal skin with multifocal pinpoint hemorrhages and oligofocal bruises in boars #1 and #2, which were euthanized on days 10 and 11, respectively (Figure 1A). In boar #1, and more severely in boar #2, the fascia spermatica externa was extensively edematous and multifocally hemorrhagic (Figure 1B). In both animals, a moderate to severe hydrocele was present, characterized by serosanguinous fluid accumulating in the processus vaginalis (Figure 1C). The proximal and distal mesorchia of boar #1 were severely edematous (Figure 1D,E) and the testicular parenchyma revealed multifocal hemorrhages (Figure 1E). In boars #2 and #3, with the latter euthanized on day 17 pi, diffuse, irregular, yellow, plaque-like lesions were present at the testicular surface (Figure 1F), whereas in boar #3, small, yellow-colored, fine filamentous adhesions were evident between the visceral and parietal layers of the tunica vaginalis, which were consistent with mesothelial proliferations (Figure 1G). Boar #4 was euthanized 25 days pi and revealed a mild serous hydrocele and fewer similar mesothelial proliferations. Furthermore, this boar had a severe suppurative prostatitis (Figure 1H).

### 3.2. Histopathology and Immunohistochemistry

Consistent with the macroscopic findings, the characteristic histopathological changes associated with ASFV infection were present in the lung, spleen, lymph node, and liver and can be found along with the immunohistochemical results in Table S2.

In the male reproductive tract, the histopathology identified up to marked alterations affecting the testis and epididymis, including the surrounding tunicae testis and accessory sex glands, but not the bulbourethral gland. The findings are summarized in Table 2.

#### 3.2.1. Testis

**Figure 2:**
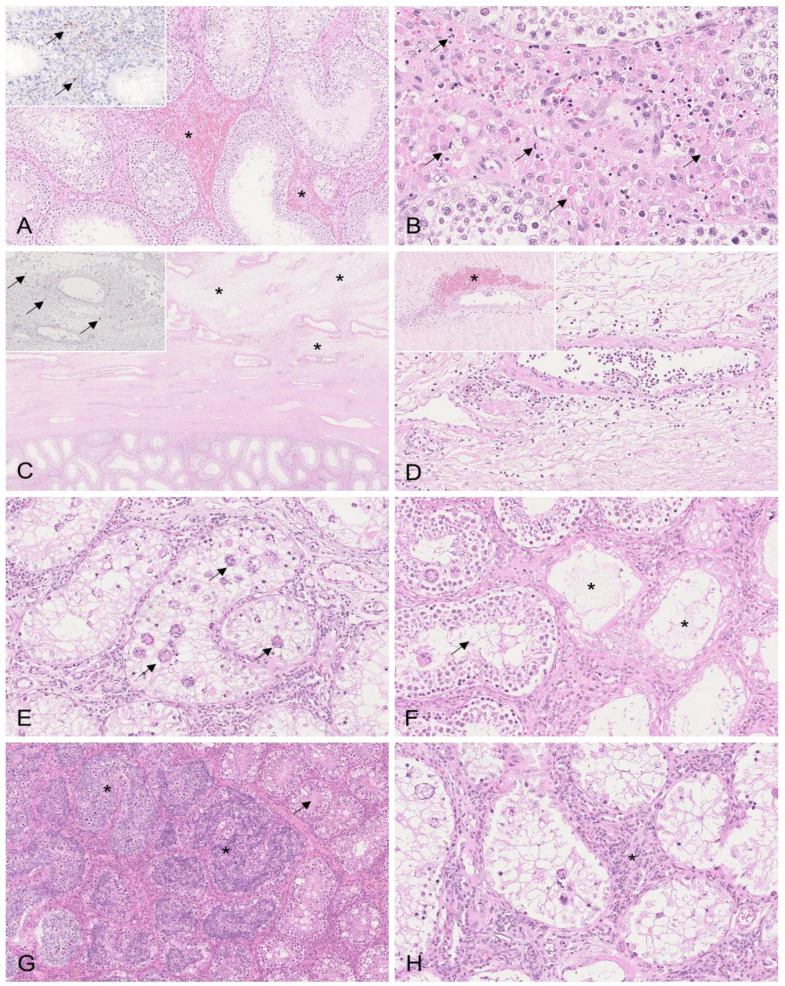
Histopathological changes to the testis in mature boars infected with moderately virulent ASFV “Estonia2014”. (**A**) HE, boar #1; hemorrhages in the testicular interstitium (asterisk). Inset: IHC, positively labeled cells (arrow) consistent with histiocytes located in the interstitium of the testis. (**B**) HE, boar #1; multifocal necrosis/apoptosis of Leydig cells (arrows). (**C**) HE, boar #1; diffuse edema expanding the tunica vasculosa and albuginea (asterisk). Inset: IHC, numerous positively labeled cells (arrow) morphologically consistent with macrophages are present in the tunica vasculosa and albuginea. (**D**) HE, boar #1; blood vessel of the tunica vasculosa with activated endothelium and transmural and perivascular infiltrates. Inset: blood vessel of the tunica albuginea with fibrinoid necrosis of the vessel wall, intramural infiltrates, and perivascular infiltrates and hemorrhage (asterisk). (**E**) HE, boar #3; seminiferous tubule with severe germ cell degeneration, epithelial vacuolation, and the formation of multinucleated giant cells (arrows). (**F**) HE, boar #4; seminiferous tubules showing different stages of degeneration with completely atrophied tubules (asterisk) adjacent to degenerating tubules with formation of single giant cells (arrow). (**G**) HE, boar #3; mildly dilated seminiferous tubules with accumulating spermatozoa and degenerating germ cells with giant cell formation (asterisk) adjacent to tubules with beginning vacuolation of germ cell epithelium (arrow). (**H**) HE, boar #3; proliferation of Leydig cells (asterisk) between degenerating seminiferous tubules.

Boar #1 showed multifocal interstitial hemorrhages of the testicular parenchyma that were consistent with the gross pathological findings (Figure 2A). In boar #1, the apoptosis/necrosis of Leydig cells was detected to different degrees (Figure 2B), as well as severe edema of the tunica albuginea, with vasculitis and/or vasculopathy affecting multiple blood vessels (Figure 2C,D). Consistent with these changes, moderate amounts of positively labeled cells consistent with histiocytes were present in the testicular interstitium between seminiferous tubules (Figure 2A, inset). Higher numbers were detected in the tunica albuginea (Figure 2C, inset). In contrast to boar #1, changes in the testis were only marginal in boar #2, and included few interstitial hemorrhages and only low amounts of immunopositive cells. The histopathological lesions were most severe in boars #3 and #4. The seminiferous tubules revealed severe degenerative changes, including germ cell degeneration and atrophy characterized by the diffuse formation of multinucleated giant cells (Figure 2E,F). Germinal epithelial cell degeneration was found along with tubular epithelial vacuolation and necrosis (Figure 2E,F). Mild tubular dilation with the impaction of spermatozoa was evident in boar #3 (Figure 2G). In addition, in boar #3, the Leydig cells were hyperplastic (Figure 2H). This boar further showed focal interstitial neutrophilic infiltration and hemorrhage. The tunica albuginea was edematous. Viral antigens were not present in either boar #3 or #4.

#### 3.2.2. Epididymis

**Figure 3:**
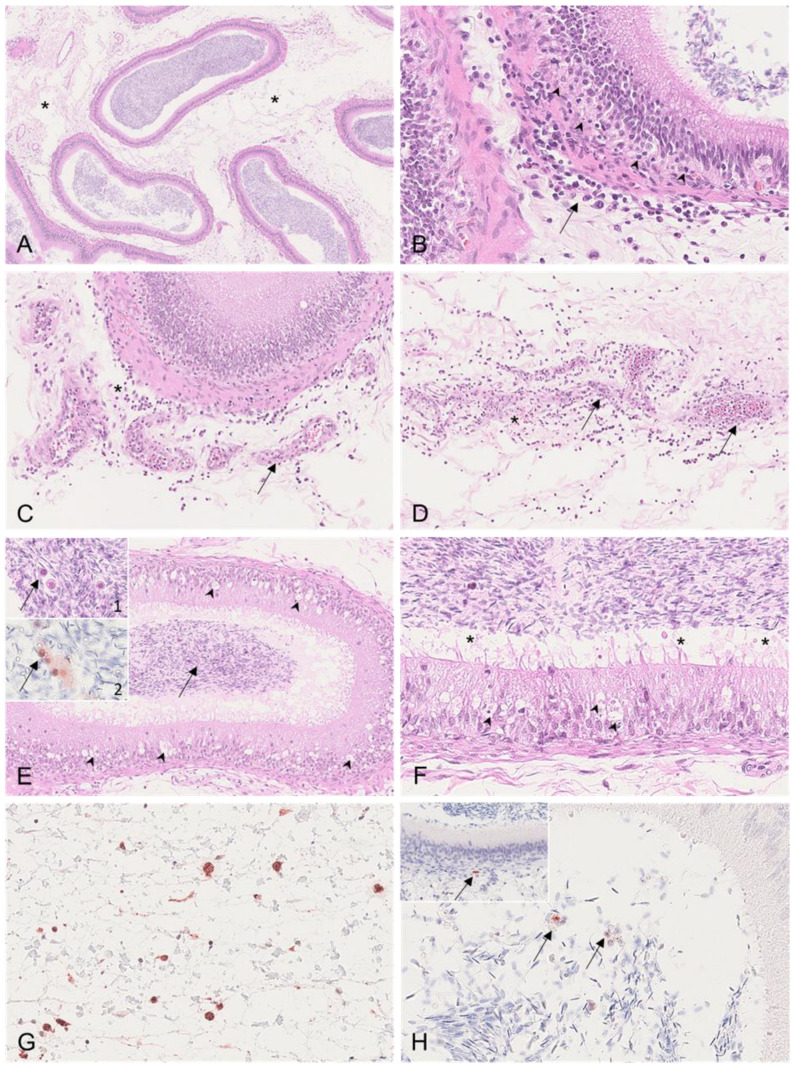
Histopathological findings of the epididymis in ASFV-infected boars. (**A**) HE, boar #1; diffuse severe edema of the epididymal head (asterisk). (**B**) HE, boar #1; the smooth muscle layer surrounding the epididymal tubules was expanded by infiltrating inflammatory cells consisting of neutrophils, histiocytes, and fewer lymphocytes (arrow). Inflammatory cells partially invaded the basal membrane (arrowhead). (**C**) HE, boar #1; necrotizing vasculitis and perivasculitis (arrow) of the epididymal interstitial vasculature adjacent to a mildly affected tubule, as shown in (**B**). The perivascular infiltrate was composed of neutrophils, histiocytes, and fewer lymphocytes (asterisk). (**D**) HE, boar #1; severe necrotizing vasculitis and perivasculitis (arrow). The tunica media and adventitia were diffusely necrotic (arrow). A focal mild hemorrhage is indicated (asterisk) within the infiltrated interstitium. (**E**) HE, boar #3; diffuse severe degeneration of the epididymal duct epithelium (arrowhead). Inset 1: Mononuclear cells were located within the tubular lumen (arrow). Inset 2: Intraluminal mononuclear cells were immunopositive for Iba-1 (arrow). (**F**) HE, boar #3; epididymal duct degeneration was characterized by cytoplasmic vacuolation, pyknosis, karyorrhexis, or karyolysis (arrowhead). Cellular debris (asterisk) admixed with spermatozoa was located within the tubule. (**G**) IHC, anti-p72, boar #1; the tunica vaginalis was infiltrated by numerous antigen-positive cells morphologically consistent with histiocytes. (**H**) IHC, anti-p72, boar #1; the lumen contained few ASFV antigen-positive cells (arrow). Inset: A single myofibroblast within the smooth muscle layer was positive for the p72 antigen.

**Figure 4:**
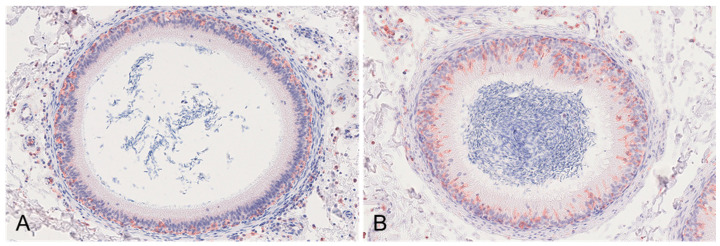
Iba-1 immunohistochemistry of epididymal tubules. (**A**) IHC, anti-p72, boar #1; Iba-1-positive macrophages were located in the basal epithelium, in the smooth muscle layer and stroma surrounding the tubule. (**B**) IHC, anti-p72, boar #3; in contrast to (**A**), immunopositive intraepithelial macrophages were found in high numbers on the basal, basolateral, and apical sites of the epithelium.

Boars #1, #2, and #3 revealed severe edema of the connective tissue of the epididymal head and body (Figure 3A) as well as mild oligofocal interstitial hemorrhages, which were observed in boars # 1 and #2. Affecting boar #1 even more, the smooth muscle layer of several epididymal tubules, predominantly affecting the head and body, was infiltrated by lymphocytes and histiocytes (Figure 3B). Further, vasculitis with perivascular lymphohistiocytic infiltration and necrosis of the connective tissue was present in boars #1 to #3 (Figure 3C,D). In contrast to boars #1 and #2, boars #3 and #4 revealed the diffuse degeneration and distinct vacuolation of the epididymal head epithelium (Figure 3E,F). In both animals, the tubular lumen was filled with spermatozoa admixed with Iba-1-positive mononuclear cells (Figure 3E, insets 1 and 2). While boar #1 revealed moderate ASFV p72 immunopositivity of cells consistent with interstitial histiocytes (Figure 3G) and, occasionally, luminal cells (Figure 3H) and myofibroblasts within the smooth muscle layer (Figure 3H, inset), the immunohistochemistry was negative in the other animals. The Iba-1 immunohistochemistry revealed marked differences between the boars euthanized early (#1 and #2) and late (#3 and #4) (Figure 4). While Iba-1-positive cells were located only to the basal site of the epididymal epithelium in boars #1 and #2 (Figure 4A), in boars #3 and #4, the positive cells increased and were also found at the basolateral and apical sites of the epithelial cells bordering the lumen (Figure 4B).

#### 3.2.3. Tunicae Testis

**Figure 5:**
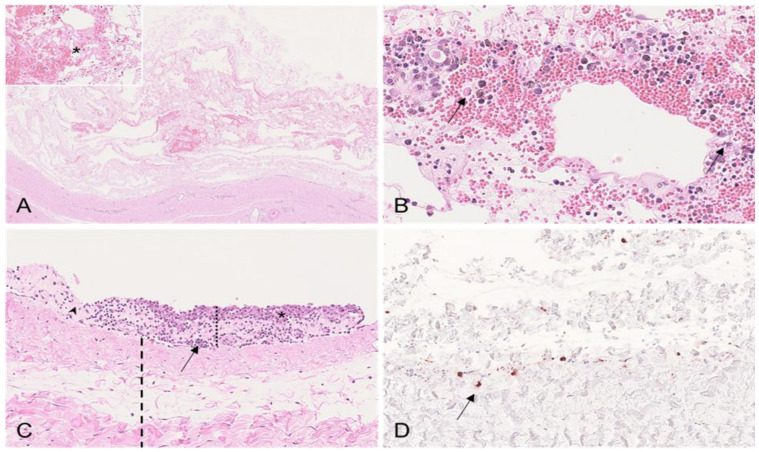
Histopathological results found in the scrotal layers in ASFV-infected boars. (**A**) HE, boar #2; severely edematous and hemorrhagic fascia spermatica externa. Inset: Fibrin deposition (asterisk) within the fascia. (**B**) HE, boar #2; hemorrhage admixed with inflammatory cells consisting of histiocytes, lymphocytes, and plasma cells in the fascia spermatica externa. There was multifocal single-cell apoptosis/necrosis of inflammatory cells (arrow), which were surrounded by multiple hemosiderin-laden macrophages. (**C**) HE, boar #2; proliferation of mesothelium (asterisk) with lymphoplasmahistiocytic infiltrates (arrow) and mild fibrous organization (arrowhead) expanding the lamina parietalis of the tunica vaginalis (black dotted line). The underlying fascia spermatica interna is illustrated by the black dashed line. (**D**) IHC, anti-p72, boar #2; demonstration of viral antigen in cells morphologically consistent with histiocytes (arrow) within the fascia spermatica externa.

The scrotal layers, or more specifically, the fascia spermatica externa and the parietal surface of the tunica vaginalis of boars #2, #3, and #4, showed histopathological changes. Boars #2 and #3 revealed severe edema of the fascia spermatica externa with hemorrhages and fibrinous deposits detected in boar #2 (Figure 5A). Furthermore, lymphohistioplasmacytic infiltrates in varying amounts were found in the fascia spermatica externa of all three affected boars (Figure 5B). Multifocally, the proliferation of the mesothelial epithelium originating from the parietal surface of the tunica vaginalis with lymphohistioplasmacytic infiltration was present in boars #2 and #3 (Figure 5C). Immunohistochemistry only showed positive results for boar #2, primarily in the fascia spermatica externa (Figure 5D). Sample material was not available for boar #1.

#### 3.2.4. Vesicular Gland

**Figure 6:**
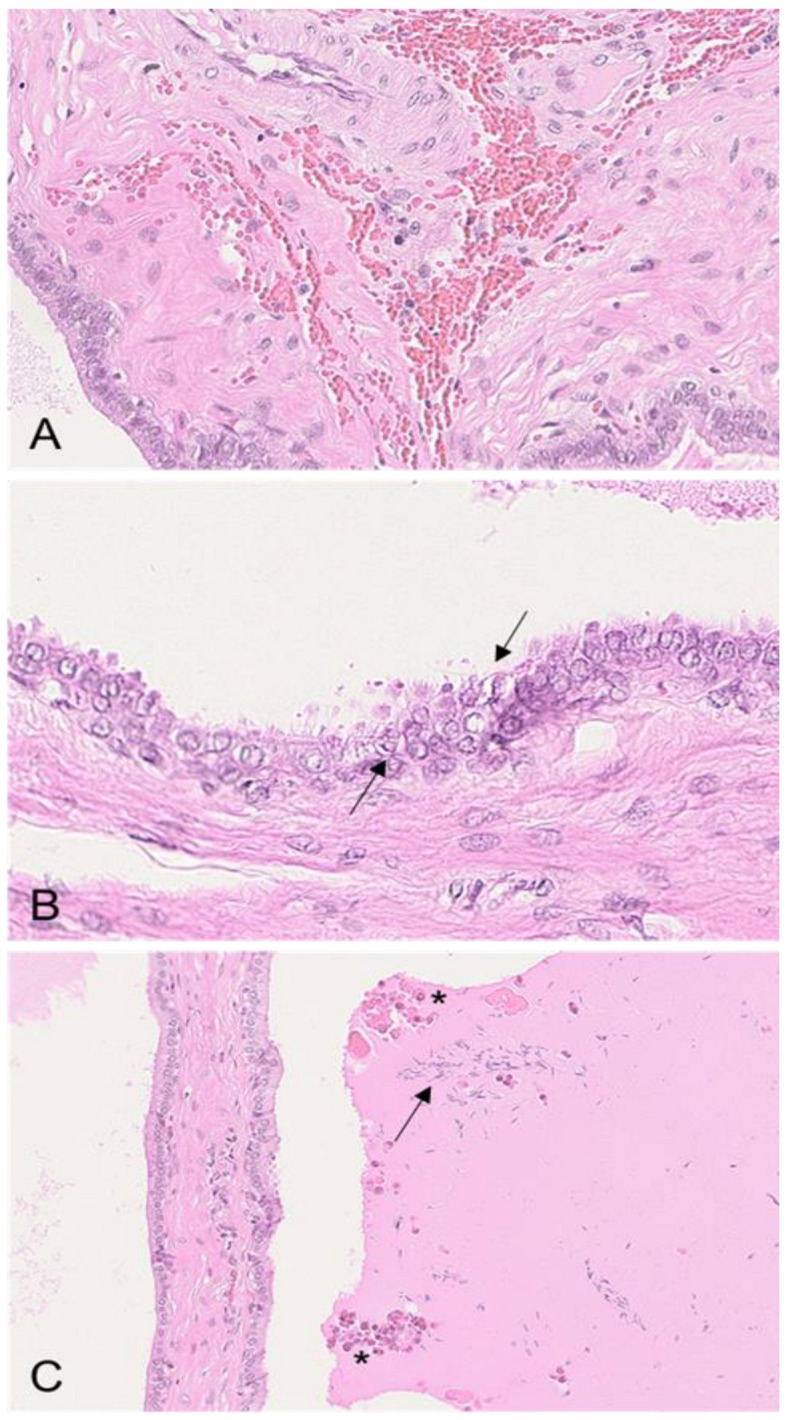
Histopathological changes in the vesicular gland of ASFV-infected boars. (**A**) HE, boar #2; hemorrhage expanding the gland interstitium. (**B**) HE, boar #4; degeneration of the glandular columnar epithelium, characterized by flattening and apoptosis/necrosis of single cells (arrow). (**C**) HE, boar #3; the lumen was filled with proteinaceous fluid admixed with cellular debris (asterisk) and several spermatozoa (arrow).

In all boars, the vesicular gland revealed only mild changes. In boar #2, oligofocal hemorrhages were found in the gland interstitium (Figure 6A). Boar #4 showed single-cell apoptosis/necrosis as well as the flattening of the gland epithelium in several areas (Figure 6B). In all boars, the luminal content was composed of proteinaceous fluid admixed with cellular debris and spermatozoa to varying degrees (Figure 6C). All boars were negative for immunohistochemistry.

#### 3.2.5. Prostate

**Figure 7:**
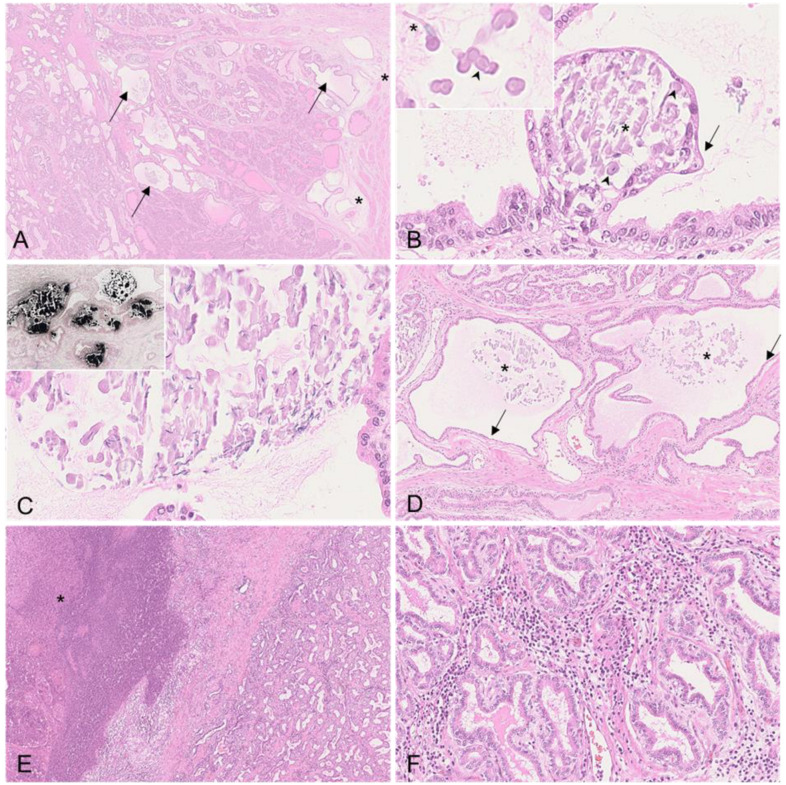
Histopathology of the prostate of ASFV-infected boars. (**A**) HE, boar #3; cystic dilation of secretory tubules (arrow) and mild edema of the fibromuscular tissue (asterisk). (**B**) HE, boar #3; vesicle formation within a secretory tubule. The vesicle was filled with sloughing epithelial cells, few spermatozoa (asterisk), and corpora amylacea (arrowhead). The vesicle was lined by a flattened, degenerating epithelium (arrow). Inset: Magnification of luminal corpora amylacea (arrowhead) and spermatozoa (asterisk). (**C**) HE, boar #3; the luminal content was largely mineralized. Inset: Mineralization of tubular content was confirmed by von Kossa stain. (**D**) HE, boar #3; in several areas, dilated secretory tubules (asterisk) were lined by flattened epithelial cells (arrow). The mineralized content is indicated by the asterisk. (**E**) HE, boar #4; suppurative-to-necrotizing prostatitis with demarcated abscess (asterisk). (**F**) HE, boar #4; interstitial infiltration by plasma cells of the remaining tissue adjacent to the abscess.

Histopathological changes were found in boars #3 and #4. In boar #3, severe dilation with vesicle formation in the secretory tubules was present (Figure 7A,B). In the secretory tubules and vesicles, proteinaceous luminal fluid was admixed with cellular debris, intermingled spermatozoa, and corpora amylacea (Figure 7B,C). These secretions were mostly mineralized (Figure 7C). In several areas, the flattening of the tubular epithelium was evident, and multifocally, the epithelial cells showed apoptosis/necrosis (Figure 7D). Mild edema of the fibromuscular tissue was prevalent (Figure 7D). In boar #4, consistent with the gross pathological findings, severe suppurative-to-necrotizing prostatitis with abscess formation was found (Figure 7A). The adjacent remaining interstitial tissue was diffusely infiltrated by plasma cells (Figure 7F). Moreover, there was also mild dilation of the secretory tubules filled with proteinaceous fluid and cellular debris. The immunohistochemistry was negative in all animals.

#### 3.2.6. Bulbourethral Gland

Neither lesions nor positive immunohistochemistry were detected in any of the boars.

### 3.3. Detection of ASFV Genome and Infectious Virus

#### 3.3.1. Semen and Semen Cell Fraction

**Figure 8:**
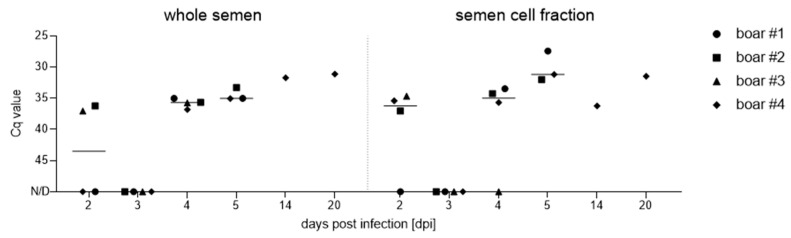
Assessment of ASFV genome loads in boar semen and corresponding cell fractions after infection with ASFV “Estonia14”. Results are depicted as Cq values and were evaluated by qPCR. Lines display the group median and each boar is represented by a unique symbol.

All detailed results regarding the detection of the ASFV genome and infectious particles in reproductive organs and semen are described elsewhere [9]. In brief, the ASFV genome could be detected at 2 dpi in whole semen (boars #2 and #3) and the semen cell fraction (boars #2, #3, and #4; Figure 8). Infectious particles were first isolated from semen starting at 2 dpi (boars #2 and #3), 3 dpi (boar #4), and 4 dpi (boar #1). All boars remained genome- and virus-positive in semen until the day of the necropsy. The infectious virus loads increased from positive to strong positive in boars #1, #2, and #3 over time, while boar #4 remained positive throughout the trial. Compared to days 2 and 4, the viral genome on day 3 was below the detection limit, likely due to the large volume of boar ejaculate (300–500 mL) and the small proportion that was subsequently used for qPCR.

#### 3.3.2. Reproductive Organs

**Figure 9:**
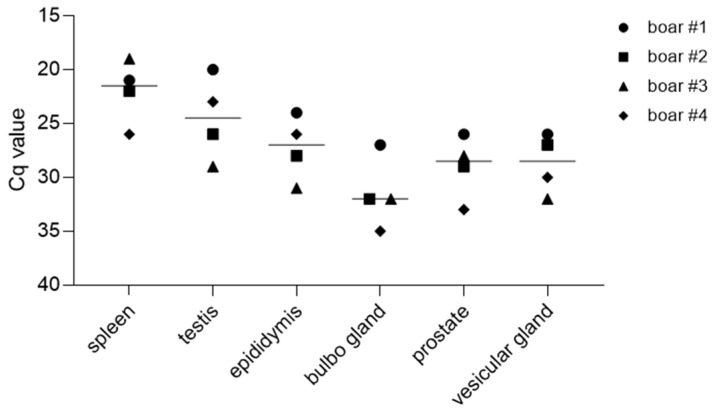
ASFV genome detection in organs of the reproductive tract of boars. Genome loads were evaluated by qPCR. Lines display the group median; each individual is represented by a symbol.

The ASFV genome was detected in all reproductive organs of the boars on the day of the necropsy (Figure 9). The mean ASFV genome loads were highest in the testes, with a mean Cq value of 24.5 ± 3.5 SD, and were lowest in the bulbourethral gland, with a mean Cq value of 31.9 ± 2.9 SD. The mean Cq values in other parts of the male reproductive tract were 27.3 ± 2.5 SD in the epididymis, 28.8 ± 2.1 SD in the vesicular gland, and 29.1 ± 2.6 SD in the prostate.

The reproductive organs obtained from the boars that were necropsied at earlier timepoints (boars #1 and #2) contained overall higher loads of the infectious virus, as evaluated by HAT (Table 3). All boars were positive in the vesicular glands and positive (boars #3 and #4) or highly positive (boars #1 and #2) in the prostate. Further, boars #2 and #3 were positive in the testes, while boar #1 was highly positive. The results for the epididymis were homogenous, with boars #1, #2, and #3 being positive. Lastly, boars #2 and #3 were weakly positive in the bulbourethral gland, while boar #1 was found to be positive. However, no infectious ASFV particles could be recovered from the testis, epididymis, or bulbourethral gland of boar #4, which was euthanized at 25 dpi. The spleen values served as reference values.

### 3.4. Assessment of Sperm Motility and Viability

**Figure 10:**
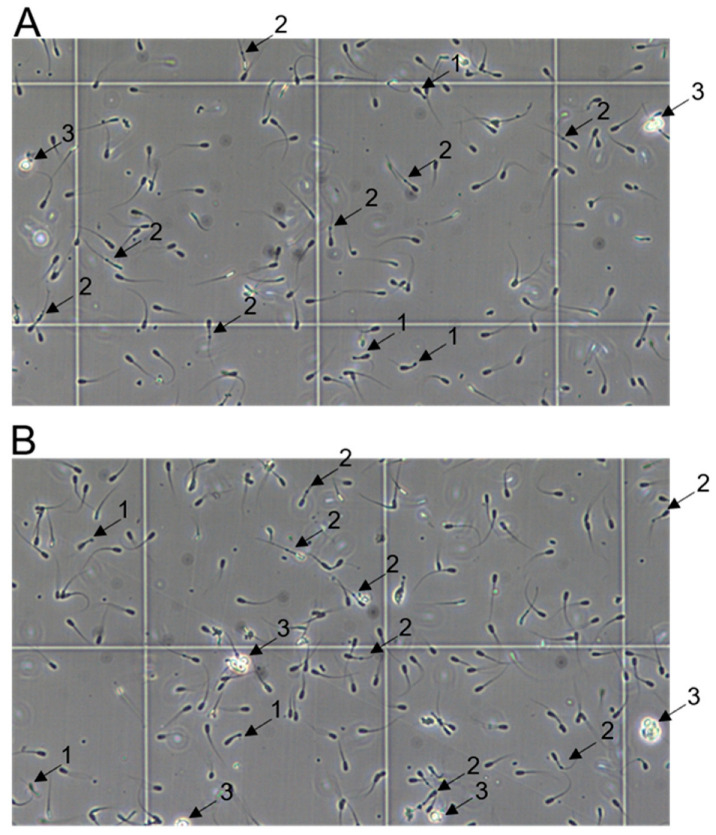
Representative sections of semen collected from boar #4 at 14 (**A**) and 20 (**B**) dpi. Abnormal spermatozoa with coiled tails (1), mobility-hampering formations at the tail (2), and round cells (3) are indicated with arrows. Viability of marked cells was validated by trypan blue staining.

An acute ASFV infection associated with a fever did not visibly affect the spermatozoa count, sperm appearance, or motility on the sampling days prior to 14 dpi, compared to −1 dpi (Table 4). However, in the samples taken at 14 and 20 dpi (boar #4), morphological abnormalities and poor motility could be observed (Figure 10). The detected morphological abnormalities mainly comprised midpiece and tail defects, e.g., aggregates at the tail and convoluted tails, ultimately hampering movement. Furthermore, consistent with the histopathological data, round cell infiltration in semen was observed at 14 and 20 dpi.

## 4. Discussion

In the present study, we succeeded in demonstrating ASFV infections of the male sexual tract using pathomorphological and virological analyses. In detail, it has been shown that an ASFV infection results in severe grossly and microscopically visible pathomorphological changes of ASFV-positive organs.

The basis of this study was the significant investigations by Roszyk et al. [7] and Friedrichs et al. [9] demonstrating that an ASFV infection not only entails infection of the reproductive organs, but can also be transmitted via semen to naïve sows. However, it has not yet been clarified at which point the virus crosses over into the sperm.

Roszyk et al. [7] clearly defined histopathological changes in the reproductive organs of acutely infected male pigs after an infection with highly virulent strains, while gross pathological changes were absent. In the testis and epididymis, necrotizing vasculitis and perivasculitis of varying degrees, as well as the apoptosis/necrosis of single stromal cells and edema, were present. The inflammation of seminiferous or epididymal tubules was only minimal, if present. Accessory sex glands, including the vesicular gland and prostate, showed only very mild changes. In parallel, in the testis and epididymis, viral antigens could be detected in high amounts. Most interestingly, immunohistochemistry identified ASFV-infected scattered epididymal halo cells, which were proposed to facilitate the disruption of the barrier integrity as the infection progressed.

In the present study, the extent of the lesions could be further examined due to the extended disease course. In contrast to the animals from the study by Roszyk et al. [7], the boars from the herein-described experiment showed obvious macroscopic lesions. Specifically, in addition to hemorrhages in the scrotal skin, the changes included edema and hemorrhages of the tunicae testis, edema of the mesorchium, hemorrhages in the testicular parenchyma, serosanguineous hydroceles, and proliferation of the mesothelium. One boar was found to have purulent prostatitis due to a secondary bacterial infection. These findings are in accordance with recently found hemorrhages in the funiculus spermaticus of a juvenile wild boar carcass [13]. While the hemorrhages and edema were caused by vascular changes [14], mesothelial proliferations in the tunica vaginalis directly represented reactive sequelae to serosal injury inflammation due to hydroceles [15]. A hydrocele is an accumulation of fluid in the scrotum between the two layers of the tunica vaginalis. In the form presented here, excessive fluid production occurred as a result either of ASFV-induced inflammation of the epididymis or testis, or generalized edema [16].

Following macroscopic examination, all organs were investigated histopathologically. The testicular lesions were comparable to those described by Roszyk et al. [7], but exceeded in severity. Interestingly, the severe degeneration and atrophy of seminiferous tubules was evident in the two boars euthanized at later time points (17 and 25 dpi). This finding is similar to testicular lesions in pigs resulting from a PRRSV infection [17]. However, because of the relatively late timing of euthanasia for animals #3 and #4, testicular lesions could not be correlated with the detection of viral antigens, but could be associated at least with the detection of viral DNA.

The changes observed in the epididymis of animals #1 and #2 were comparable to those previously described by Roszyk et al. [7], with the corresponding detection of viral antigens at least in boar #1. Strikingly, and similarly to the testis, boars #3 and #4 showed highly degenerative changes in the epididymal head and body, which were characterized by vacuolation and the necrosis of the tubular epithelium. Of note, high numbers of semen round cells were identified in these two animals. Mononuclear cells must be distinguished from germinal epithelial cells, which are shed into the lumen in testicular degeneration [18]. In the present study, these cells were clearly identified as macrophages by immunohistochemistry against ionized calcium-binding adaptor molecule 1 (Iba-1) [19]. Macrophages contribute to first-line immune defense and immune surveillance in the male reproductive tract [20]. Semen macrophages originate from the testicular interstitium [21]. In men, to a certain concentration, semen macrophages are considered to be physiologic; however, in pigs, mononuclear cells should not be present in the adluminal compartment of the testicular or epididymal tubules [17]. Compared to those in the testis, macrophages in the epididymis are not well characterized [22]. However, an increase in macrophages, as seen in these boars, could represent an enhanced immune response, which would be supported by the severe lesions found in the testis and epididymis. Similarly, intraluminal mononuclear cells have also been detected in PRRSV-infected pigs at 27 or 28 dpi, while these were absent in uninfected control pigs [17]. However, viral antigens could not be demonstrated in macrophages from either PRRSV-infected or ASFV-infected pigs in this study, so the source of infection or transition of the virus into sperm has yet to be elucidated.

Significant changes were also present in the prostates of animals #3 and #4, which were associated with the detection of the viral genome and infectious virus. Corpora amylacea were found in cystically dilated tubules, which are thought to be related to epithelial cell degeneration and desquamation [23]. Further, luminal mineralization was evident, which has been suggested to be the result of the calcification of the corpora amylacea [24,25]. With regard to changes in the prostate, it must be mentioned that boar #4 showed purulent inflammation. Since several organs of this animal were affected, the ASF was probably complicated by a systemic bacterial secondary infection, as is usually seen after an infection with ASFV strains of a lower virulence [26]. Further, intraluminal spermatozoa were identified in both the prostate and vesicular gland, which could be indicative of an ejaculatory reflux from the urethra back to the accessory glands. This phenomenon has also been described for seminal vesiculitis in bulls. The detection of spermatozoa in the accessory sex glands was suspected to be associated with an asynchrony of neurophysiological processes in the ducts of the accessory sex glands during ejaculation due to inflammation [18].

As the aim of the initial study was the collection of ASFV-infected sperm, no mature control pigs were included and were therefore not available for histopathology. However, when evaluating pathogen-related changes, it is crucial to distinguish background lesions in respective organs. According to one study, testicular tubular and epididymal atrophy can occur with increasing age in miniature pigs [27]. Although those lesions have been insufficiently documented in conventional domestic swine breeds so far, the herein-described lesions should be interpreted with caution at this point due to the low number of animals examined.

Based on the present findings, including the severe macroscopic and histopathological changes correlated with positive virus detection, both in the reproductive organs and in fresh ejaculate samples, it can be concluded that an ASFV infection leads to the breakdown of the blood–testis barrier (BTB) and blood–epididymis barrier (BEB). Since epididymal sperm was sampled and examined only postmortem, and contamination with infected blood could not be excluded, as shown by Roszyk et al., this question has not yet been conclusively clarified. The BTB is one of the tightest barriers in mammals. It is located among Sertoli cells and between Sertoli and germ cells, dividing the seminiferous tubule into basal and adluminal compartments [28]. The adluminal compartment is separated from the circulatory and lymphatic system, allowing spermatocytes and spermatids to complete meiosis. Likewise, the BEB is formed by tight junctions between epithelial cells of the epididymal ducts, favoring the proper maturation of spermatozoa in an immune-privileged environment [29]. Inflammatory lesions of the interstitium, including infiltrates or edema, as seen in ASFV-infected pigs, are major causes of barrier breakdown due to a massive release of inflammatory cytokines [30,31]. This eventually leads to leukocyte infiltration into the interstitium, as well as into seminiferous and epididymal epithelia [32,33], as observed in the present study. Further, a compromise in the barrier results in changes to sperm motility and viability [32], which were also demonstrated in ASFV-infected boars.

The proven venereal transmission of ASF disastrously illustrates the risk of rapid spreading to a plethora of susceptible pigs. Although the risk of transmission in commercial swine holdings is expected to be low through early culling, this problem can become uncontrollable in backyard herds and particularly in the wild boar population [34,35]. Friedrichs et al. [9] accurately showed that the libido and fertility of boars is not reduced by an ASFV-induced illness, resulting in the solid shedding and transmission of the virus via semen. In particular, this scenario is conceivable in the case of the circulation of moderately virulent strains, which is not unlikely in the course of emerging virus variants [36].

## 5. Conclusions

The aim of the present study was to investigate pathological changes in the male reproductive tract caused by a protracted ASFV infection, and thereby to identify the route of ASFV entry into semen. Beyond the acute stage of the disease, ASFV-infected pigs revealed severe lesions in the testis and epididymis, but also in the accessory sex glands, involving a morphologically visible breakdown of the blood–testis and blood–epididymis barriers. The destruction of the blood–testis and blood–epididymis barriers must, however, have taken place beforehand, since the semen of infected boars was already positive for viral DNA starting on day 2 after infection. At this point, more in-depth research is needed to identify and characterize the crosstalk of ASFV infections, inflammation, and the cellular response of the barrier microenvironment in the testis and epididymis.

## Figures and Tables

**Table 1:** Gross pathological findings of reproductive organs detected in ASFV-infected mature domestic breeding boars.

	Boar #1	Boar #2	Boar #3	Boar #4
Day of humane endpoint	10	11	17	25
Scrotum	Scrotal skin with hemorrhages; serosanguinous hydrocele testis		-	Serous hydrocele testis
Testicle	Parenchymal hemorrhages; edema of mesorchium	Mesothelial proliferations		
Tunicae testis	Hemorrhage; edema		-	
Prostate	-			Suppurative prostatitis

**Table 2:** Histopathological findings of reproductive organs detected in ASFV-infected mature domestic breeding boars.

		Boar #1	Boar #2	Boar #3	Boar #4
Day of humane end-point		10	11	17	25
Testis	HE	Interstitial hemorrhages Single-cell apoptosis/necrosis of Leydig cells Edema of tunica albuginea Vasculitis/vasculopathy	Interstitial hemorrhages	Germ cell degeneration/atrophy with formation of multinucleated giant cells Germinal epithelial degeneration with vacuolation of tubular epithelium Tubular epithelial necrosis Tubular dilation Hyperplasia of Leydig cells Interstitial infiltration with neutrophils Interstitial hemorrhage Edema and congestion of tunica albuginea	Germ cell degeneration/atrophy with formation of multinucleated giant cells Germinal epithelial degeneration with vacuolation of tubular epithelium Tubular epithelial necrosis Edema and congestion of tunica albuginea
	IHC	3	1	0	0
		Interstitial histiocytes Tunica albuginea			
Tunicae testis	HE	N/A	Hemorrhagic edema of fascia spermatica externa with fibrin Lymphohistioplasmacytic infiltrates in fascia spermatica externa Mesothelial proliferations	Hemorrhagic edema of fascia spermatica externa Lymphohistioplasmacytic infiltrates in fascia spermatica externa Mesothelial proliferations	Lymphohistioplasmacytic infiltrates in fascia spermatica externa
	IHC	N/A	2	0	0
		N/A	Fascia spermatica externa, histiocytes		
Epididymis	HE	Head/body: Intertubular edema Interstitial hemorrhages Peritubular infiltration with histiocytes/lymphocytes Vasculitis/vasculopathy	Head/body: Intertubular edema Interstitial hemorrhages Perivascular infiltration of lymphocytes and histocytes	Head/body: Intertubular edema Perivascular infiltration with lymphocytes and histiocytes Vacuolation and degeneration of tubular epithelium Tail: Intertubular edema Peritubular infiltration with lymphocytes and histiocytes Tunica vaginalis: Perivascular infiltration of lymphocytes and histiocytes Fibrovascular projections	Head/body: Vacuolation and degeneration of tubular epithelium
	IHC	2	0	0	0
		Interstitial histiocytes Myofibroblasts Luminal round cells			
Vesicular gland	HE	Luminal content with cellular debris and spermatozoa	Interstitial hemorrhages Luminal content with cellular debris and spermatozoa	Luminal content with cellular debris and spermatozoa	Single-cell apoptosis/necrosis of glandular epithelium Luminal content with cellular debris and spermatozoa
	IHC	0			
Prostate	HE	-	-	Dilation of prostatic tubules Luminal content with cellular debris, spermatozoa, and corpora amylacea; mineralized Flattening of tubular epithelium with single-cell apoptosis/necrosis Edema of fibromuscular tissue	Suppurative prostatitis Dilation of prostatic tubules Luminal content with cellular debris Interstitial infiltration with plasma cells
	IHC	0			
Bulbourethral gland	HE	-	-	-	-
	IHC	0			

**Table 3:** Hemadsorption test results for reproductive organs of boars.

	#1	#2	#3	#4
Testis	+++	++	++	−
Epididymis	++	++	++	−
Bulbourethral gland	++	+	+	−
Vesicular gland	++	++	++	++
Prostate	+++	+++	++	++
Spleen	+++	+++	−	++

− = negative; + = weak positive; ++ = positive; +++ = strong positive.

**Table 4:** Total numbers of spermatozoa counted and proportions of normal and abnormal spermatozoa observed in each ejaculate after inoculation with ASFV.

dpi	Boar #	Normal	Abnormal	% Abnormalities
2	1	108	13	10.7
	2	167	13	7.2
	3	143	10	6.5
	4	100	7	6.5
3	1	211	26	11
	2	208	20	8.8
	3	129	15	10.4
	4	242	21	8
4	1	113	16	12.4
	2	247	28	10.2
	3	107	11	9.3
	4	148	17	10.3
5	1	117	19	14
	2	172	24	12.2
	3	—	—	—
	4	180	21	10.4
14	4	139	29	17.3
20	4	247	51	17.1

## Data Availability

Data are available from the corresponding author upon request.

## Supplementary Materials

The following supporting information can be downloaded at: https://www.mdpi.com/article/10.3390/v15030729/s1, Table S1. Gross pathology evaluation results of boars infected with ASFV “Estonia 2014”; Table S2. Histopathological findings in boars infected with ASFV “Estonia 2014”; Figure S1. Summary of macroscopical findings in boars infected with the moderately virulent ASFV strain “Estonia2014”.
