# Supplementary material for: Pathology of African Swine Fever in Reproductive Organs of Mature Breeding Boars
Source: Viruses. 2023 Mar 11;15(3):729. doi: 10.3390/v15030729 (PMC10055891; doi:10.3390/v15030729)
Supplement: Supplementary file 1 [file viruses-15-00729-s001.zip › Figure S1.docx]

**Figure S1**. Summary of macroscopical findings in boar infected with the moderately virulent ASFV strain “Estonia2014”. Stacked bar diagram depicting the total gross lesion score. Lesions were evaluated on a scale from 0 to 3.
